# 

**Published:** 1876-08

**Authors:** 


### Table of Contents.

### ORIGINAL COMMUNICATIONS.

Stone in the Bladder.... 257
Esmarch's Bloodless Operation.. 263
An Inch Screw Swallowed by a Child Two Years Old.... 264

### ATLANTA ACADEMY OF MEDICINE.

Premature Delivery—Therapeutic Action of Ammonia—Quinine in Typhoid Fever—Tumor Extracted from Orbit of Eye—Case of supposed Perityphlitis—Treatment of Typhoid Fever.... 264

### OUR EXCHANGES.

A Case of Post-Diphtheritic Paralysis.... 277
Concerning the Inoculability of Typhoid Fever.... 279
Remedy for Dysmenorrhœa.... 280
The Enemata of Wine.... 281
The Most Useful Drugs.... 282
Application of Quinia in Fever....
Treatment of Rheumatic Fever with Salicylic Acid.... 283
Section of United Fracture and Wiring of the Ends.... 284
On the Employment of Cold.... 285
Camphor Poisoning.... 286
Etherization.... 287
When we May, and When we May Not, Bleed.... 288
Treatment of Epistaxis by Internal Administration of Ergot....
Whooping Cough.... 289
Cod-Liver Oil as a Therapeutic Agent.... 291
Salicylic Acid as a Febrifuge.... 292
Treatment of Syphilitic Buboes.... 293
Syphilis—Is it Ever a Cause of Phthisis?.... 294
Treatment of Cystitis by Atropia Enemata.... 295
Gelseminum Sempervirens....
Injection of Quinine in Gonorrhœa.... 296
Chloral in the Pains of Parturition....
Scientific Careers.... 297
Tetanus Following the Hypodermic Injection of Quinine.... 298
Extraordinary Success in Ovariotomy.... 299
Can Tuberculous Matter Taken as Food Excite Tuberculous Disease?
Treatment of Orchitis.... 300
Arrest of Convulsions by the Sinistro-Lateral Posture.... 301
Cholera Morbus.... 303
Monthly Abortions.... 305

### BIBLIOGRAPHICAL.

Lectures on Orthopedic Surgery and Diseases of the Joints.... 305
A Treatise on the Diseases of the Nervous System.... 309
Micro-Photographs in Histology, Normal and Pathological.... 310
Pamphlets Received....

### EDITORIAL.

The Convention of Medical Colleges.... 312
International Medical Congress.... 314
The American Dermatological Association.... 315
Dr. R. D. Arnold, of Savannah.... 316
Dr. Edward Pharr.... 319
Flint River Medical Association....

